# A Comparative Study of Anterior Segment Optical Coherence Tomography and Gonioscopy in Detection of Narrow, Occludable Angles

**DOI:** 10.7759/cureus.69879

**Published:** 2024-09-21

**Authors:** Abhay Lune, Apurva Prabhudesai

**Affiliations:** 1 Ophthalmology, Dr. D. Y. Patil Medical College, Hospital and Research Centre, Dr. D. Y. Patil Vidyapeeth, Pune (Deemed to be University), Pune, IND

**Keywords:** as oct, glaucoma, gonioscopy, narrow angles, preservation of vision

## Abstract

Introduction

Glaucoma is a group of progressive optic neuropathies and is a leading cause of blindness. Primary angle-closure glaucoma is a global health problem, and if left untreated, it can lead to irreversible vision loss. Anterior segment imaging with optical coherence tomography (AS-OCT) is a rapidly advancing field in ophthalmology. Early detection of narrow, occludable angles using AS-OCT can lead to early and prompt management, resulting in the preservation of vision.

Methods

We conducted a cross-sectional, outpatient department (OPD)-based study of 212 eyes of patients. Eyes with narrow angles were selected for this study. Eyes identified as having narrow angles on Van Herick (grade ≤2) were confirmed by gonioscopy, and the angles were graded according to the Shaffer gonioscopy grading system. These eyes were further tested using AS-OCT, and the angles, measured in degrees, were graded according to the degrees given in the Shaffer anterior chamber angle grading system. The grades obtained on AS-OCT were compared with those obtained on gonioscopy. The correlation between the narrow angles measured on AS-OCT and those measured on gonioscopy was determined.

Results

The mean age of the participants was 58.64 years. Of the 212 eyes found to have narrow angles on gonioscopy, 206 eyes (97.2%, 95% CI: 94.0%-99.0%) also had narrow angles on AS-OCT, while six eyes (2.8%, 95% CI: 1.0%-6.1%) were found to have open angles. The chi-square test for association yielded a p-value of <0.001, indicating a statistically significant relationship between AS-OCT and gonioscopy grading.

Conclusion

This study showed a high correlation between AS-OCT and gonioscopy for grading narrow angles. Gonioscopy remains the gold standard for detecting and managing narrow angles. AS-OCT can be used to identify narrow angles in busy OPDs where these cases can easily be missed. These patients can then be further evaluated with gonioscopy and managed if necessary.

## Introduction

Glaucoma is defined as a group of progressive optic neuropathies that cause changes in the optic nerve head by destroying retinal ganglion cells and the retinal nerve fiber layer. Glaucoma is the second most common cause of blindness worldwide and the leading cause of irreversible blindness [1].

Primary angle-closure glaucoma (PACG) has the worst prognosis among all types of glaucoma. Approximately 23 million people are affected by PACG, and this number is expected to increase to 32 million by 2040 [2]. PACG is estimated to affect 26% of all glaucoma patients and is responsible for 50% of blindness caused by glaucoma [3]. The Indian population has a substantial risk of PACG. In one study, 34.5% of glaucoma patients had primary angle-closure disease, and 40% of eyes with glaucoma had advanced disease [4]. Another study found that more than 90% of PACG patients were unaware of their condition [5]. Additionally, in patients with primary angle closure, 24.8% had acute angle closure, a serious vision-threatening condition [6].

In PACG, early detection of narrow, occludable angles can lead to prompt management and preservation of vision. Anterior chamber angle (ACA) evaluation is important in diagnosing and treating glaucoma and plays a role in its prevention. Although slit-lamp gonioscopy is considered the gold standard for ACA evaluation, its poor reproducibility and long learning curve present limitations. Rapid, objective screening methods are needed to identify eyes at risk [7].

Anterior segment optical coherence tomography (AS-OCT) is a non-invasive, non-contact technology based on the principle of low-coherence interferometry. With AS-OCT, cross-sectional and 3D images of the ACA can be obtained with high resolution. Both qualitative and quantitative analysis can be performed. The test is faster and does not require the level of expertise needed for gonioscopy.

AS-OCT imaging is a rapidly advancing field in ophthalmology. The images can be stored and compared with larger databases. They can also be classified automatically based on the database, which may facilitate the assessment of narrow angles and the associated risk of developing PACG.

Previous studies have reported AS-OCT sensitivity in detecting narrow angles ranging from 73% to 98%. We conducted this study in western India to determine the correlation of AS-OCT with gonioscopy in patients with narrow angles and to assess whether it can be routinely used in a busy outpatient department (OPD) to identify patients at risk of angle closure.

## Materials and methods

This cross-sectional study was conducted at a tertiary healthcare center in western India between July 2022 and 2024. Approval was obtained from the institutional ethics committee for the study protocol, and informed consent was obtained from all participants before enrollment.

In this study, we included all previously undiagnosed glaucoma patients and patients over the age of 40, as the prevalence of primary angle closure disease (PACD) increases with age. Patients younger than 40 years of age and those with a previous diagnosis of glaucoma were excluded. Patients with media opacities, such as corneal edema and opacity, nuclear sclerosis, a history of trauma or previous eye surgery, and those with systemic illnesses were also excluded. We selected 212 eyes of patients who matched the inclusion criteria.

Each patient underwent a comprehensive eye examination, including uncorrected visual acuity, refraction, and best-corrected visual acuity (BCVA), as well as a detailed slit-lamp evaluation that included Van Herick's test, intraocular pressure assessment using Goldman applanation tonometry, and an undilated fundus evaluation by 90D and/or a direct fundus ophthalmoscope evaluation of the central fundus, macula, and optic nerve head. Eyes with narrow angles on the Van Herick test (grade ≤ 2) were subjected to gonioscopy, followed by further analysis using AS-OCT.

Goni oscopy was performed on all patients. The investigator informed them about the procedure, and one drop of topical anesthetic was instilled into the eye. Gonioscopy was conducted in a room with low lighting and minimal slit-lamp illumination. We used the Goldman four-mirror lens for gonioscopy with the eye in the primary position of gaze, taking care to avoid applying pressure to the eye with the goniolens.

A coupling agent (viscoelastic) was applied to the goniolens' contact surface, and the patient was asked to look up before the goniolens was inserted into the lower fornix. The patient was then asked to look straight ahead, ensuring that no light fell over the pupillary plane. The slit width was reduced to minimal height and width with low intensity. Shaffer's gonioscopy grading classification was utilized to grade and classify each eye's temporal quadrant.

For all patients, AS-OCT was performed in a dark room. The OCT machine used in this study was the Zeiss Cirrus 500 HD OCT with an anterior segment attachment. The procedure was explained to the patients beforehand. After properly aligning each patient's right eye, we took cross-sectional images of the irido-corneal angle (temporal quadrant) of the anterior chamber, commonly known as the anterior chamber angle. The scleral spur was identified in the angle images.

Gonioscopy angles, based on the structures visible in the anterior chamber angle at the temporal quadrant, were converted into grades according to Shaffer’s grading system. The angle was defined as the angle between the posterior corneal surface and the anterior iris surface. Closed or slit angles, where no structures were visible, were considered grade 0. Extremely narrow angles, where only Schwalbe's line was visible, were considered grade 1 (1-10 degrees). Narrow angles, where the trabecular meshwork was visible, were considered grade 2 (11-20 degrees). All eyes with grades 0, 1, and 2 were classified as having narrow angles. Angles with visible scleral spur and ciliary body were considered open, with grade 3 (21-30 degrees) and grade 4 (31-40 degrees or more).

In this study, we used AS-OCT to take cross-sectional images of the anterior chamber angle. We identified the angle structures, namely the scleral spur, posterior corneal surface, and anterior iris surface, required for measuring the angle in degrees. We manually marked the apex of the angle, the posterior surface of the cornea, and the anterior surface of the iris, and then used the caliper software tool to calculate the ACA in degrees.

The AS-OCT measured anterior chamber angles in degrees. The angles measured on AS-OCT were matched to Shaffer's grading system for angle conversion as follows: angles measured as 0 on AS-OCT were considered closed, grade 0; angles from 1 to 10 degrees were considered extremely narrow (grade 1); angles from 11 to 20 degrees were considered narrow (grade 2); angles from 21 to 30 degrees were considered open (grade 3); and angles from 31 to 40 degrees or more were considered open (grade 4).

We used Shaffer's grading system to classify the angles as 0 (closed or slit), 1 (extremely narrow), 2 (narrow), and 3 and 4 (open angles) (Table 1). We compared the grades of angles obtained on gonioscopy with those obtained on AS-OCT using the Shaffer grading system.

**Table 1:** Angle conversion of AS-OCT to gonioscopy grading using Shaffer’s angle grading in degrees AS-OCT: anterior segment optical coherence tomography.

Shaffer’s angle grading in degrees	Gonioscopy grading	AS-OCT angle in degrees	AS-OCT grading
0	0	0	0
1-10	1	1-10	1
11-20	2	11-20	2
21-30	3	21-30	3
31-40 or more	4	31-40 or more	4

Statistical analysis was performed using IBM SPSS Statistics for Windows, Version 17 (Released 2008; IBM Corp., Armonk, New York). The normal distribution of data was assessed using the Shapiro-Wilk test. Qualitative variables, such as age and gender, were presented as proportions. The sensitivity of AS-OCT in detecting narrow angles, based on the anterior chamber angle in degrees, was compared against gonioscopy using Shaffer's grading for detecting narrow angles. The proportions, along with a 95% confidence interval, were reported.

## Results

This study included 212 eyes with narrow, occludable angles that fulfilled the inclusion criteria. Of these, 121 (57.1%) eyes belonged to men and 91 (42.9%) belonged to women. In the 40- to 60-year-old age group, 74 eyes (60.7%) were from males and 48 eyes (33.3%) were from females. Among participants aged >60 years, 47 eyes (52.2%) were from males and 43 eyes (47.8%) were from females. The mean age was 58.64 years, with a range of 43 to 76 years.

The mean intraocular pressure (IOP) measured with applanation tonometry (AT) was 16.12 mm Hg, with a minimum of 12 mm Hg and a maximum of 23 mm Hg. According to Van Herick’s grading system, 20 eyes (9.4%) were in grade 0, 128 eyes (60.4%) were in grade 1, and 64 eyes (30.2%) were in grade 2.

The gonioscopy results in (Table 2) show the distribution of grades among the observed cases. Grade 0, where no structures were seen, was observed in 30 cases, accounting for 14.2% of the total. Grade 1, where the angle was open up to Schwalbe's line (SL), was noted in 59 cases, making up 27.8% of the total. Grade 2, where the angle was open up to the trabecular meshwork (TM), comprised 123 cases or 58.0% of the total.

**Table 2:** Eyes with narrow angles in study group, classified according to Shaffers gonioscopy grading system Eyes with grade 0 and slit were grouped together in grade 0. Eyes with grades 3 and 4 (open angles) were not included in the study.

Gonioscopy	Angle in degrees	Number of eyes	Percentage of eyes
Grade 0: Closed and Slit	0	30	14.2
Grade 1: Schwalbe’s line visible	10	59	27.8
Grade 2: Trabecular meshwork visible	20	123	58.0
Grade 3: Scleral spur visible	30	0	0
Grade 4: Ciliary body visible	40	0	0
Total	-	212	100.0

The distribution of AS-OCT grades showed a varied distribution across each grade. Grade 0 was found in 10.4% of the total, and grade I in 30.7%. Grade II was the most common, comprising 56.1%, while grade III was found in 2.8% of the total (Table 3).

**Table 3:** Eyes with AS-OCT grading in study group AS-OCT: anterior segment optical coherence tomography.

AS-OCT grade	Number	Percentage
Grade 0	22	10.4
Grade 1	65	30.7
Grade 2	119	56.1
Grade 3	6	2.8
Total	212	100.0

**Figure 1:**
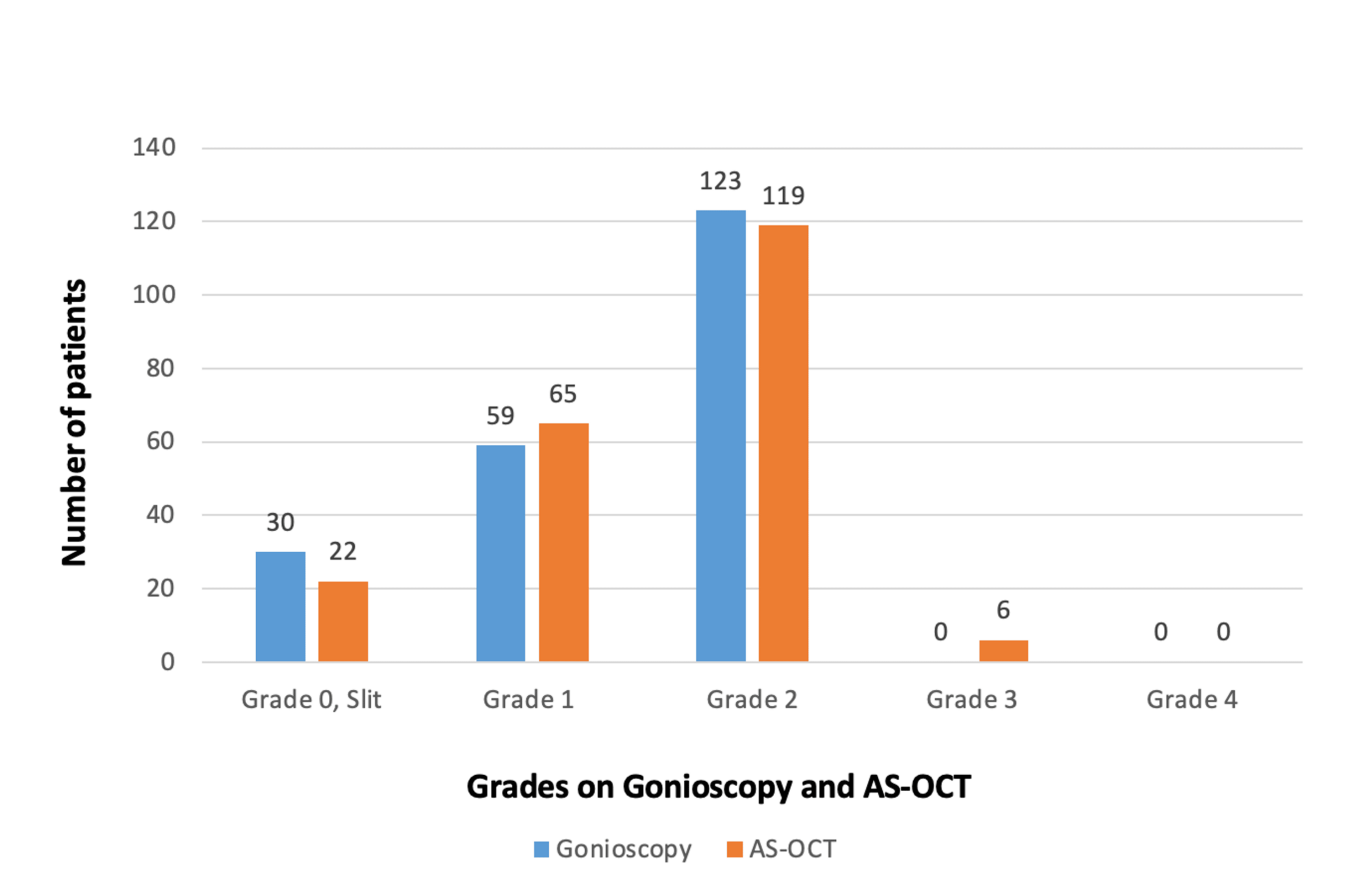
Comparison of anterior chamber angle grades on gonioscopy and AS-OCT Grade 0 was observed in 30 patients on gonioscopy and in 22 patients on AS-OCT. Grade 1 was observed in 59 patients on gonioscopy and in 65 patients on AS-OCT. Grade 2 was observed in 123 patients on gonioscopy and in 119 patients on AS-OCT. Grade 3 was seen in 6 patients on AS-OCT. AS-OCT: anterior segment optical coherence tomography.

Grade 0 was observed in 30 patients on gonioscopy and in 22 patients on AS-OCT. Grade 1 was observed in 59 patients on gonioscopy and in 65 patients on AS-OCT. Grade 2 was observed in 123 patients on gonioscopy and in 119 patients on AS-OCT. Grade 3 was seen in six patients on AS-OCT. Eyes with grades 3 and 4 on gonioscopy were not included in the study group (Figure 1).

Exact grade-wise correlation between gonioscopy and AS-OCT revealed that, of the 30 eyes with grade 0 on gonioscopy, 22 (73.3%) were grade 0 and eight (26.7%) were grade 1 on AS-OCT. Of the 59 eyes with grade 1 on gonioscopy, 50 (84.8%) were grade 1 and nine (15.3%) were grade 2 on AS-OCT. Of the 123 eyes with grade 2 on gonioscopy, 110 (89.4%) were grade 2, and 13 (10.6%) eyes did not match; seven (5.7%) were grade 1, and six (4.9%) were grade 3 on AS-OCT (Table 4).

**Table 4:** Correlation between AS-OCT and gonioscopy grading AS-OCT: anterior segment optical coherence tomography, N: number of eyes.

Grades	Gonioscopy (N)	Eyes correlated with AS-OCT and gonioscopy grading, N (%)	Eyes that did not correlate between AS-OCT and gonioscopy grading, N (%)
0	30	22 (73.3%)	8 (26.7%)
1	59	50 (84.8%)	9 (15.3%)
2	123	110 (89.4%)	13 (10.6%)
Total	212	182 (85.85%)	30 (14.15%)

AS-OCT angle measurements for closed and slit angles in degrees in the study cohort showed a mean of 0 degrees. For extremely narrow angles, the mean was 8.57 degrees, and for narrow angles, the mean was 14.97 degrees. AS-OCT angle measurements for all angles in the study cohort showed a mean of 12.098 degrees. Out of the total 212 eyes with narrow angles on gonioscopy, AS-OCT correctly identified 206 (97.2%) eyes with narrow angles (Table 5).

**Table 5:** Diagnostic accuracy of AS-OCT compared to gonioscopy in detecting narrow angles

Correlation between gonioscopy and AS-OCT	Gonioscopy	Percentage
Correlation present between gonioscopy and AS-OCT for narrow angles	206	97.2
No correlation present between gonioscopy and AS-OCT for narrow angles	6	2.8
Total	212	100.0

The chi-square test for association yielded a p-value of less than 0.001, indicating a statistically significant relationship between AS-OCT and gonioscopy grading. Cohen's kappa score was 0.84, indicating near-perfect agreement between AS-OCT and gonioscopy grading. The sensitivity of AS-OCT was 97.2% (95% CI: 94.0%-99.0%). Only six eyes (2.8%) showed an open angle (grade III) on AS-OCT (95% CI: 1.0%-6.1%).

## Discussion

We conducted this cross-sectional study to compare gonioscopy and AS-OCT and to determine whether there is any significant correlation between the narrow angles visualized on gonioscopy and the narrow angles measured by AS-OCT. Based on Van Herick's grading system and gonioscopy, we included 212 eyes with narrow angles in our study. In the study group, 121 (57.1%) eyes belonged to males, and 91 (42.9%) to females. The mean age was 58.64 years, with a range of 43-76 years.

Van Herick’s grading system for angle grading revealed 20 eyes (9.4%) with grade 0, 128 eyes (60.4%) with grade 1, and 64 eyes (30.2%) with grade 2. Shaffer’s gonioscopy grading system showed grade 0 in 30 eyes (14.2%), grade 1 in 59 eyes (27.8%), and grade 2 in 123 eyes (58.0%). Regarding the distribution of AS-OCT anterior chamber angle grades, grade 0 was seen in 22 (10.4%) eyes, grade 1 in 65 (30.7%), grade 2 in 119 (56.1%), and grade 3 in six (2.8%) eyes.

We correlated Shaffer's grading (0, 1, and 2) for narrow angles exactly with gonioscopy and AS-OCT. Of the 30 eyes with grade 0 on gonioscopy, 22 (73.3%) had grade 0 on AS-OCT, while eight (26.7%) were graded as grade 1. Of the 59 eyes with grade 1 on gonioscopy, 50 (84.8%) had grade 1, and nine (15.3%) had grade 2 on AS-OCT. Of the 123 eyes with grade 2 on gonioscopy, 110 (89.4%) had grade 2, and 13 (10.6%) did not match on AS-OCT. Of these 13 eyes, seven (5.7%) had grade 1, and six (4.9%) had grade 3 on AS-OCT.

Wirbelauer et al. conducted a study using slit-lamp-based OCT for non-contact goniometry with optical coherence tomography. Sensitivity was calculated for the detection of occludable angles with a gonioscopy grade of less than or equal to 2. The discriminant threshold for OCT goniometry was determined as an anterior chamber angle lower than 22 degrees. The sensitivity of OCT goniometry (AS-OCT) to detect an occludable angle was 86% for the anterior chamber angle [8].

Nolan et al. studied primary angle closure using AS-OCT in Asian eyes. They reported that AS-OCT had excellent sensitivity (98%) in detecting angle closure compared to gonioscopy. They concluded that AS-OCT is a rapid, non-contact method of imaging angle structures and is highly sensitive in detecting angle closure compared to gonioscopy [9]. In a study conducted by Lavanya et al. on a Southeast Asian population, AS-OCT showed a sensitivity of 88.4% [10]. Källmark and Sakhi evaluated nasal and temporal anterior chamber angles using various techniques and concluded there was good agreement between gonioscopy, the Van Herick technique, and AS-OCT [11].

Dabasia et al. conducted a study on non-contact screening methods for detecting narrow anterior chamber angles. They concluded that the Van Herick test and OCT ACA (ACA < 20.78 degrees) were the best techniques for distinguishing between narrow and open angles. It was concluded that ACA < 20.78 degrees could be used to identify individuals who might benefit from further gonioscopic testing in a screening setting [12]. Kochupurakal et al. conducted a study to assess the role of AS-OCT in detecting narrow angles. They evaluated the trabeculo-iris angle (TIA), which is the same as the ACA, and determined a cutoff value of TIA ≤ 22 degrees for detecting narrow angles on AS-OCT. The sensitivity of AS-OCT for detecting narrow angles was 90.7% for TIA. It was concluded that the sensitivity of AS-OCT was high for detecting narrow angles compared to gonioscopy [13].

Porporato et al. concluded that AS-OCT offers a quick, user-friendly, and objective non-contact method of assessing the anterior segment and angle, which is well-tolerated by patients and correlates well with the information provided by gonioscopy. They found good evidence that AS-OCT has diagnostic accuracy in detecting angle closure [14]. Khan et al. assessed the anterior chamber angle using SD-OCT in a normal population in central India. A comprehensive eye examination was done along with gonioscopy. AS-OCT was performed to evaluate the angle, and patients with narrow angles on gonioscopy (less than grade 2 on Shaffer’s grading) had TIA values of less than 20 degrees on OCT. Despite gonioscopy being the gold standard for angle evaluation, AS-OCT provides a rapid, non-contact, and quantitative method [15]. Jaseena et al. conducted a study in South India comparing gonioscopy and AS-OCT to assess the anterior chamber angle. They found the diagnostic effectiveness of AS-OCT to be moderate compared to gonioscopy, with a sensitivity of 73% [16].

Imaging of the anterior chamber angle with AS-OCT has certain limitations. Direct visualization of the angle for assessing pigmentation or neovascularization is not possible as only a cross-sectional image is obtained. Additionally, the superior and inferior quadrants are difficult to scan with AS-OCT, and manipulation may lead to distortion of the angle [17].

In our study, only 2.8% (six eyes) were classified as having open angles (grade 3) on AS-OCT imaging. Although classified as open, these eyes had angle measurements ranging from 21 to 24 degrees, indicating they were close to the cutoff value of 20 degrees. As the anterior chamber angle anatomy can change over time due to physiological changes in lens morphology, these eyes require close observation and periodic follow-up. The remaining 97.2% (206 eyes) were correctly identified as narrow on AS-OCT.

There are several limitations in our study. The main limitation is that only patients with narrow angles were included. We compared the correlation between AS-OCT grading and gonioscopy. A single investigator performed the AS-OCT measurements for all patients.

As there is no established classification for angle grading in degrees for ACA measurement on AS-OCT, based on the studies mentioned above, we used ACA <20 degrees as the cutoff value for narrow angles on AS-OCT [12,15].

Another limitation is that other practical aspects, such as cost-effectiveness, ease of use, and clinical applicability, were not considered in this study. Future studies are needed to assess the feasibility of AS-OCT in clinical practice. Further research is also necessary to evaluate whether AS-OCT can be used as an adjunct to gonioscopy for angle evaluation in clinical settings.

## Conclusions

Our study compared AS-OCT to gonioscopy solely in terms of grading correlation for narrow angles. The AS-OCT anterior chamber angle grading demonstrated a high correlation with gonioscopy grading. Gonioscopy remains the gold standard for the management of glaucoma. Further research is needed to assess the overall feasibility and practicality of AS-OCT, as well as to evaluate its broader clinical application as a screening tool.
